# Video games and disability—a risk and benefit analysis

**DOI:** 10.3389/fresc.2024.1343057

**Published:** 2024-03-01

**Authors:** Hung Jen Kuo, Michael Yeomans, Derek Ruiz, Chien-Chun Lin

**Affiliations:** ^1^ Department of Counseling, Educational Psychology and Special Education, Michigan State University, East Lansing, MI, United States; ^2^ Rehabilitation and Disability Studies, Southern University and A&M College, Baton Rouge, LA, United States; ^3^ Rehabilitation and Mental Health Counseling, Western Oregon University, Monmouth, IL, United States

**Keywords:** video games, disability, rehabilitation, risks, benefits, self-determination theory, motivation

## Abstract

**Purpose:**

Over the past decades, video games have become a substantial part of the entertainment industry. While ubiquitous, video game participation remains low among people with disabilities amid potential negative effects. This article analyzes the risks and benefits that video games may present to individuals with disabilities.

**Methodology:**

In this conceptual article, we explored the literature pertaining to video games and disability. To better understand the impact of video games on individuals with disabilities, we focused on the unique features of video games through the lens of the Self-Determination Theory.

**Findings:**

Our findings show that individuals with disabilities are most at risk from excessive video game use, leading to increased aggression, sedentary behavior, and negative impact on academic performance. Identified benefits include promoting physical rehabilitation and psychological well-being, improving cognitive abilities and emotional regulation, and utility in promoting exercises, and managing chronic pain.

**Originality:**

This article presents a number of strategies and resources to help guide individuals with disabilities, educators, practitioners, and researchers in maximizing the benefits of video games while controlling the risks.

## Video game and disability—a benefit and risk analysis

The video game industry has grown exponentially over the past few years, and there are no signs of slowing down (1). The revenue generated by the video game industry is more than that of movies and sports combined (2). As Pallavicini et al. (3) mentioned, video games have become a common daily activity for most adults, and the video game population is equally distributed across gender and age. The number of people playing video games is even more robust because of the COVID-19 pandemic, during which other forms of entertainment, such as movies and live sports events, were restricted (4).

While video games seem ubiquitous, not everyone has equitable access to them. A plethora of literature points out that individuals with disabilities have lower participation in video games [e.g., (5–7)]. Individuals with disabilities typically encounter two major challenges when playing video games. First, not all video games are accessible. As Grammenos et al. (7) described, video games have higher or unique demands for individuals’ motor, sensor, and mental skills. For example, in some games, players need to press the exact button(s) on a controller at a specific time to achieve the objectives. Super Mario Bros series, as an example, require players to control the main character to run and jump precisely to pass the level. The precision of hand-eye coordination and short response times needed for mastering the games subsequently influence the overall playing experience. In addition, individuals with cognitive impairments may find it challenging to navigate games with puzzle quests, wherein players must fully comprehend and follow the instructions to achieve the objectives (8). Compulsive use is another common concern that may hinder individuals with disabilities from engaging with video games healthily. This concern is particularly true for younger individuals with developmental disabilities. For example, Mazurek and Engelhardt (9) indicated that some believe playing certain types of video game leads to certain behavioral problems, especially those with developmental disabilities such as autism spectrum disorder The reported issues range from inattention (10) and oppositional behaviors (9) to video game addictions (11, 12).

Despite these concerns, researchers continue to invest in what video games can offer for people with different abilities. Some studies used video games as a motivator to promote academic learning [e.g., (13–15)]; to improve cognitive abilities [e.g., (16, 17)]; and to enhance physical strength [e.g., (18, 19)]. The undeniable benefits, in conjunction with potential risks, have yielded much discourse supporting and against the use of video games in educational and clinical settings. Whether to leverage the utility of video games or altogether avoid them becomes a result of personal choice and clinical judgment of educators, practitioners, and individuals.

### Purpose

Considering the contentious nature of video games, this article examined studies published in the past two decades that explore the relationship between individuals with disabilities and their engagement with video games. Specifically, we searched databases, including Google Scholar, ProQuest, EBSCOhost, and ResearchGate, with keywords including disability, rehabilitation, video games, and gamification. For a more focused search, we set the search limitation to include only peer-reviewed journal articles and articles written in English. Although we attempted to capture as fully as possible, readers should be aware that this article is not a systematic review of the literature but a summarization of what we discovered from the literature. Therefore, interpreting our findings and suggestions should be cautious.

In this article, our primary aim is to illustrate that, despite being controversial and raising certain concerns, video games can serve as a valuable tool due to their unique features for motivating engagement. We argue that whether to play video games is not a simple all-or-none decision; instead, the key rests on making players with disabilities aware of video game benefits and risks while facilitating their informed decisions. In addition, we seek to draw attention from practitioners, researchers, and educators to leverage video games as valuable tools and advocate for equitable access to the digital entertainment world for individuals with disabilities. In this article, we (a) discuss the evolution of video games, (b) discuss the uniqueness of video games from a theoretical standpoint, (c) explore the risks and benefits associated with video games, and (d) provide practical strategies and resources for leveraging video games that maximize the benefits while controlling the risks.

## The evolution and uniqueness of video games

As the digital world evolves, video game technology has also advanced from traditional two-joystick and one-monitor entertainment to a more sophisticated and immersed player experience. To date, gaming consoles such as PlayStation and Xbox offer a much higher resolution picture (4 K) with vibrant colors, significantly improving players’ immersive experience. In addition, various control mechanisms have been introduced to allow for real-life simulation, such as a steering wheel for driving games and simulated instruments for music games. The evolution of virtual reality technology pushes a step further for a more immersive experience by submerging users in a three-dimension world. To accommodate individual preferences in content and style of play, video game genres are many and varied, including action, adventure, role-playing, puzzle, simulation, strategy, and sports (details for each type of video games can be found in Table 1). A single-player game is optimal for individuals who enjoy playing the game by themselves; for those who enjoy playing with friends, cooperative games (or “co-op games”) can be played. If the person prefers to play cooperative games but cannot find anyone nearby, an online game can be an option. More recently, accessibility features were built natively in some games to assist individuals with various disabilities. For instance, high-contrast color and captioning can be enabled to assist individuals with sensory impairments, and button remapping and adaptive controller can be useful for those with finger dexterity challenges. The abundance of options satisfies various preferences and abilities, thereby providing a great deal of freedom for users to choose whatever, however, and whenever they would like to play these games.

**Table 1:** Video game type.

Game type	Descriptions	Examples
Action games	Action games prioritize physical challenges, hand-eye coordination, and reaction time. Players typically navigate levels, fight enemies, and complete tasks that require swift reflexes.	• Super Mario Bros • Devil May Cry
Adventure games	Adventure games prioritize story-driven experiences, puzzle-solving, and exploration. To advance through a storyline, players usually engage with characters and objects.	• The Legend of Zelda • Life is Strange
Role-playing games (RPGs)	In RPGs, players can become characters in make-believe worlds. These games often feature character development, storytelling, and decision-making that have an impact on the game's outcome.	• Final Fantasy • Elden Ring • Mass Effect
Simulation games	Simulation games imitate real-world activities or systems. Players engage in tasks ranging from managing cities and farms to flying airplanes or driving vehicles.	• The Sims • Microsoft Flight Simulator
Strategy games	Strategy games require players to plan and make decisions to achieve specific goals. They can be turn-based or real-time, involving resource management, tactics, and sometimes warfare.	• Civilization • StarCraft • Age of Empires
Sports and racing games	Sports games simulate real-world sports, while racing games focus on competitive racing experiences. These games often feature realistic physics and gameplay mechanics.	• FIFA • Gran Turismo • Mario Kart
Fighting games	Fighting games involve one-on-one combat between characters, each with unique moves and abilities. Players compete to deplete their opponent's health bar.	• Street Fighter • Mortal Kombat
Horror games	Horror games aim to create a sense of fear and suspense. They often involve survival elements and focus on storytelling to immerse players in a tense atmosphere.	• Resident Evil • Silent Hill
Massively multiplayer online role-playing games	MMORPGs are online multiplayer games where large numbers of players interact in a persistent virtual world. They often involve character progression, social interaction, and cooperative play.	• World of Warcraft • Guild Wars 2
Puzzle games	Puzzle games challenge players with logic-based problems and tasks. They can range from simple puzzles to complex brain teasers.	• Tetris • Portal

## Theoretical perspective—self-determination theory

**Figure 1:**
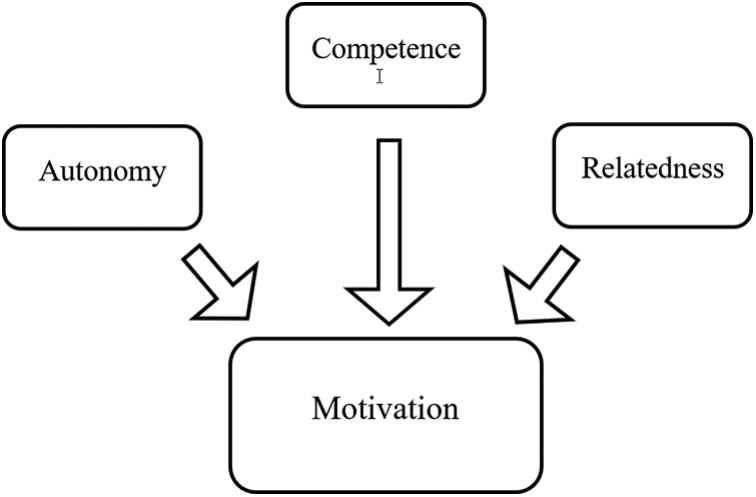
Self-Determination theory. The three elements, autonomy, competence, and relatedness, that drive people's motivation in self-determination theory.

The freedom of choice, which gives players a sense of control, is perhaps best understood from the *self-determination theory* [SDT; (20)]. This empirically derived theory depicts the psychological needs of an individual when engaging in an activity. The satisfaction of these psychological needs leads to either motivating or discouraging continue engagement of the activities. When these psychological needs are satisfied, the individual feels empowered by self-determination. On the other hand, when the needs are somewhat deprived, the individual may choose, if allowed, to avoid such activities. These psychological needs can be categorized into three dimensions: autonomy, competence, and relatedness (20) (see Figure 1). Autonomy pertains to an individual's volition for engaging in specific tasks. Therefore, the individual may do certain things voluntarily or, in extreme cases, be coerced. In the context of playing video games, as Ryan et al. (21) described, almost all cases are voluntary; therefore, the need for autonomy is easily satisfied.

Competence pertains to one's need to be challenged, providing a sense of achievement. Based on effort justification theory, Inzlicht and colleagues (22) explained that an individual's effort toward a given pursuit would add value to its outcome. Therefore, completing an easy task may not be as satisfying as completing a challenging task. In the video game sphere, players usually start a game from an easier level, and by design, most games increase the level of challenge as they progress. As players progress in the game, the sense of achievement becomes a natural gaming experience.

Relatedness pertains to the need for belonging or connecting with others. According to Ryan et al. (21), video games effectively meet this need from two perspectives. First, computer-generated personality and artificial intelligence can produce a sense of belonging when players are immersed in the games. For example, in a role-playing game (RPG), players may immerse themselves in the stories and befriend non-player characters (NPCs). Another source for the sense of relatedness can be found in multiplayer or online games. As Hsu and Lu (23) described, the online game community provides players with a sense of group cohesiveness and, in turn, positively affects players’ loyalty and motivation to the game.

The capacity of video games to satisfy an individual's psychological needs of self-determination is a double-blade sword, however. For example, a sense of achievement obtained from passing a game level can be uplifting, but if the pursuit of such a feeling gets in the way of daily life, it becomes a problem. We believe that only by understanding the risks and benefits of video games, we will be able to help individuals with disabilities make informed choices. To this end, we discuss the risks and benefits associated with video games in the following section.

## Risks of video games

The available literature on the risks of video games examines their relationship to excessive use, their associations with aggressive and sedentary behavior, and the academic problems that can develop because of overuse.

### Excessive use of video games

It should be noted that excessively playing video games differs from other forms of addiction. Video games, unlike substances such as alcohol and drug, do not directly manipulate neurotransmitters or alter brain functioning. Continuing to play video games is typically motivated by in-game rewards (24). In the context of SDT, it can be understood that playing video games satisfies psychological needs that are otherwise difficult to achieve. Typically, an individual would fulfill self-determination needs across various life events such as education, work, and recreation. Hence, a good balance can be maintained. However, when playing video games becomes the dominating source of self-determination need, it becomes problematic (25). Due to its impacts, problematic video game behavior and pathology have caught much attention lately (24).

In addition, the diagnosis standards, such as the 11th revision of the International Classification of Diseases (ICD 11), have also included gaming disorder as a formal diagnosis, defining it as a gaming behavior of sufficient severity to result in significant impairment in areas of function. Similarly, the Diagnostic and Statistical Manual of Mental Disorders-Text Revision (DSM-5 TR) also identifies Internet Gaming Disorder (IGD) as a condition that requires clinical attention (26). A recent study conducted by Masi et al. (27) reported children with attention-deficit/hyperactivity disorder (ADHD) are vulnerable to excessive use of video games compared. Similarly, in a survey of 23,533 adults, Andreassen et al. (28) found that ADHD, obsessive-compulsive disorder (OCD), anxiety, and depression are associated with problematic use of social media (15%) and video games (7%).

However, the prevalence and the etiology of problematic use of video games may be debatable (29). In a comprehensive review, Przybylski and colleagues argued that the prevalence rate may not be accurate as relevant studies do not distinguish between passionate engagement and pathological symptoms. Specifically, Przybylski et al. (30) indicated that of those who play games, more than two in three did not report any symptoms of IGD. Furthermore, they also suggested that only a small proportion of the general population—between 0.3% and 1.0%—may qualify for a potential acute diagnosis of IGD. Similarly, although pathological internet use (PIU) is commonly observed in those who play online games (31), it is questionable to attribute the problem strictly to video games. For example, researchers argued that the “gaming” component of the video game does not cause PIU. Instead, it is the interactive feature, such as online chatting and messaging, which generates a sense of companionship that causes the problem (32).

### Aggressive behavior

Compared to more passive media such as television and movies, the interactive nature of video games may increase the likelihood of aggressive behavior by allowing players to engage in behaviors that are not otherwise socially accepted in the real world (33). About 90% of the games contain some form of violent content, and 40% include serious violence against other characters (34). This is alarming because several studies have found connections between violent video games and delinquent or violent behaviors (35, 36). Specifically, an analysis of 3,372 Flemish adolescents revealed that violent video gaming was positively related to individual delinquent behavior. In contrast, nonviolent video gaming was not found to be related to problematic behaviors, suggesting that the content of video games matters (36). Similarly, Anderson and his team (2010) reported that violent video game content was positively related to aggressive behaviors, thought patterns, affect, and a lack of prosocial behaviors (e.g., empathy) in real life.

However, while some research suggested that violent content in video games may promote higher levels of aggressive behaviors in real life, others argued that the strength of its influence is debatable. For example, in a study of youth in eighth and eleventh-grade students, DeCamp and Ferguson (35) noted that violent video games were a weak predictor for violent behaviors compared to other predictors (e.g., home environment, relationship with parents, and demographics). Specifically, factors such as poor family relationships and prior abusive experience were far more critical regarding youth violent behaviors.

### Sedentary behaviors

Sedentary behaviors, like watching television and playing video games, are highly prevalent in youth and may be associated with physical and mental health markers. In a systematic review, Kontostoli et al. (37) observed that the time children and adolescents spent playing video games limited their time for healthier physical activities, raising health concerns such as cardiovascular disease (CVD). Similarly, in a study of Korean adolescents, Byun and colleagues (38) found that participants’ time spent watching TV or playing video games was associated with the risk of high adiposity. Consistent results were reported in European and American children and adolescents (39, 40). Recognizing this potential issue, video game technology has evolved creatively to battle it. For example, some games would prompt players to rest after a continuous playing period and those that require players to interact with full-body movements.

### Poorer academic performance

In the United State, it is reported that 60% of video gamers are below 34 years old (41). Of these gamers, 40% are below 18. Due to the ubiquitous video game engagement among the young population, concerns regarding the impact of video games on academic performance are common. One longitudinal study conducted by Jackson et al. (42) sought to examine whether the time spent on Internet use and video game playing influenced the academic performance of 12-year-old youths for a year. As a result, video games were found to be negatively correlated with academic performance. However, this relationship only existed among those who had a lower GPA to begin with. Importantly, Jackson et al. (42) noted that using GPA as a single measure may be misleading, particularly for young children. For example, playing video games was found to be beneficial to these children's development of visual-spatial skills. However, these skills may not yet be part of the academic requirement at the 6th or 7th-grade levels; thereby, the GPA did not reflect the benefit. Nevertheless, the negative effect of playing video games on academic performance was echoed by many others across different age groups [e.g., (43–45)].

## Benefits of video games

Despite the many risks associated with video games, many benefits have also been reported. For example, video games can be a vehicle for enhancing cognitive abilities, increasing emotional regulation, managing chronic pain, and promoting physical activity.

### Cognitive abilities enhancement

The utility of video games to enhance human ability can be traced back to 30 years ago when researchers first used them to teach children communication and spatial abilities (46). The highly customizable content and interactive feature make video games unique when used as a training tool. Game designers can embed learning content in the game and provide immediate rewards when learners complete a required task. These features, from the lens of the SDT, really help promote the autonomy and competence needs of the users, and ultimately motivate users to continue their engagement. Leveraging this unique feature, researchers have also used video games to train people with disabilities. For example, video games were found to be effective for developing social skills in children with dyslexia, learning difficulties, and autism spectrum disorder (ASD) (47–50). In a recent study, Beaumont and colleagues found that children with ASD can benefit from video game-based social skills training, especially with parental involvement. Similarly, in a systematic review, Eichenberg and Schott (51) aptly indicated that video games, when infused with cognitive behavioral techniques, are valuable for improving behavioral, cognitive, and emotional outcomes of those with learning and cognitive disabilities.

In addition, Ashinoff and Abu-Akel (52) demonstrated using video games to improve the attention span among those with ADHD. Specifically, they explained that when individuals with ADHD play video games, their alpha and beta levels in the frontal lobe reduce. This reduction in alpha and beta levels signifies that the individuals can maintain their engagement in a given task effortlessly, hence achieving a hyperfocus status. Although the generability still needs testing, it shows promise for maintaining attention in the ADHD population.

### Serious gaming

There is a plethora of research conducted on using video games for educational purposes [e.g., (53, 54)]. These are usually referred to as serious games, which are designed with educational goals, such as science, mathematics, languages, history, spatial problem solving, logic, and mental rotation skills while providing engaging, interactive, and fun learning environments for single or multiple players (53, 54). The utility of serious gaming has caught much attention in K-12 classroom settings, both mainstream and special education, for its unique features that create authentic learning situations, promote intrinsic motivation, and interactions to promote social learning and individualized learning (55). Serious games can be found in many settings, such as the military, medicine, and manufacturing (56).

For training individuals with disabilities, serious gaming can be helpful as well. For example, Papanastasiou et al. (55) reviewed previous studies and suggested that serious games positively influence the executive functioning of students with intellectual and developmental disabilities, ADHD, and ASD. Specifically, they argued that multi-sensory stimuli can accommodate students with different learning styles. From the lens of the SDT, video game-infused learning can promote autonomous learning, thereby motivating students’ engagement. Papanastasiou et al. (55) reported that this type of learning can yield positive outcomes, including enhanced perception, attention and cognition, phonological skills, visual-spatial attention, increased social engagement, and independence. A more recent study also demonstrated positive outcomes from playing serious games, which results in improved attention, time management, and planning/organizing, and decreased hyperactivity symptoms among students with ADHD and learning disabilities (57).

### Emotional regulation

Emotional regulation is the ability to monitor, evaluate, and control one's emotions. Emotional regulation is associated with several important outcomes, including psychological well-being, relationship satisfaction, and overall mental health (58, 59). In contrast, emotional dysregulation, which is commonly observed in ASD, ADHD (60, 61), and the chronic pain populations (62, 63), is associated with many forms of psychopathology and maladaptive behaviors (64).

In a systematic review, Villani et al. (65) found that video games have a lot to offer regarding emotional regulation. First, video games provide players opportunities to actively interact with the game, which directly influences their locus of control and sense of autonomy—a critical element of the SDT. Additionally, the exposure to negative emotional stimuli during gameplay (e.g., frustration during a difficult level in the game) offers an opportunity for players’ ability to be aware of their emotional state (66, 67) and build resilience to frustration (68). Importantly, Gaggioli et al. (69) and Tunney et al. (70) argue that video games can be instrumental in training emotional regulation because of their highly customizable features. For example, gradually adjusting the game difficulty levels to be harder and harder can be useful for building frustration tolerance.

Lastly, video games can also help people cope with stressors such as job loss (71) and bereavement (72). In a survey study, Iacovides and Mekler (73) explored the relationship between video games and their effect on players’ coping mechanisms. Specifically, their attention was on whether the video game would help them escape temporarily from real-life distress. While escapism is often considered a maladaptive coping mechanism, the study argued that adequate escapism can be a healthy distraction that is much needed for some. As Kuo et al. (74) further explained, healthy escapism represents a temporary shift of attention from the self, which can be achieved by projecting oneself onto in-game characters. This escapism allows players to explore versions of their ideal or aspirational selves, thereby fostering a better self-understanding.

### Chronic pain management

Chronic pain is a common symptom shared by people with various physical disabilities (75). It is associated with many negative outcomes, such as poor goal attainment (76), school performance (77) and social functioning (78). Chronic pain is also associated with higher rates of anxiety (79), insomnia (80), and depressive symptoms (81).

As early as the 1980s, Redd and colleagues (82) found that video games were an effective way to distract attention and manage pain. Since then, its applications for pain management have been widely used. For example, video games were found to be effective pain relief for patients going through unpleasant medical treatments such as chemotherapy (83, 84). Additionally, occupational therapists and physical therapists have used video games with burn patients (85) as a means of distracting from pain as well as rehabilitating physical strengths (86, 87). To this end, video games can be a non-pharmaceutical complementary intervention and can be easily integrated with existing medical procedures and therapies.

### Increasing physical activities

Maintaining a regular exercise habit for a healthy lifestyle can be challenging for anyone. Willson et al. (88) used the SDT to explore factors influencing individuals’ motivation to maintain exercise. In their analysis, although each element of the SDT interacts with the exercise motivation differently and may depend on the individual (e.g., females have a different competence needs than males for exercises), their influences are without question. Interestingly, as mentioned earlier, video games bear the capacity for fulfilling all three needs of the SDT. Therefore, using video games to motivate physical exercise can be an effective application.

Active Video Games (AVGs), also referred to as “exergames,” consist of games that require players to exert physical energy to progress through the game (89). AVGs have been used in occupational and physical therapy settings for over 30 years. These game-based exercises are helpful for task-specific rehabilitation that aims to enhance function in hands and extremities (90, 91), movements, balance, and mobility after brain injury (92–94). In addition, research also shows that some games may improve physical fitness for wheelchair users with spinal cord injuries, nerve diseases, and multiple sclerosis (95, 96). Despite a small sample size, Singh et al. (97) demonstrated that playing interactive VR games would increase reaction time in adults with disabilities.

Aside from rehabilitative goals, AVGs provide an opportunity to engage in physical activities that otherwise might not be possible due to social distancing or other life commitments such as work and school (89). This increase in physical activity, in turn, can reduce depressive symptoms and improve mental health (98). Studies also show that participation in exercise games positively affects self-esteem and self-efficacy and may promote intrinsic motivation to continue exercise outside of video game play (99, 100).

## Strategies and resources

It is clear that playing video games can yield both risks and benefits. The question is then how to maximize the benefits while controlling the risks. In the following section, we provide some strategies for leveraging video games’ benefits and unique features. Specifically, we suggest to (a) form a well-defined purpose for playing video games, (b) be cautious of content and disability populations that are associated with risks, and (c) monitor and supervise the use of video games. Lastly, we recognize the fast evolving pace of video game technology. It is becoming challenging to stay updated without reliable sources of information. To this end, we would like to introduce a list of useful online video game resources that specifically aim to assist individuals with disabilities to engage in video games.

### Well-defined purpose

People choose to play video games for several reasons, such as entertainment, education, social connection, rehabilitation, or any combination of these. While video games were originally designed to entertain players, their utility has since expanded. Although there can be numerous reasons for playing video games, it is helpful for players to identify the primary reason for playing them in a particular setting. As Nebel et al. (101) emphasized, explicit goal-setting would improve video game experiences by increasing motivation and lowering cognitive load compared to those without a well-defined goal. Similarly, Brusso et al. (102) highlighted the importance of realistic goal-setting to individual performance in video game-based training. Therefore, if the reason for playing video games is not strictly for entertainment, a well-defined and explicit goal should be delineated to set realistic expectations and accountability. A well-defined goal would inform the outcome evaluation and monitoring, and help decide the types of games and the ways of interacting with them. For example, if the intent is to do more exercises or physical rehabilitation, AVGs (e.g., Just Dance, Ring Fit Adventure) would be ideal; if the individuals would like to enhance decision-making and critical thinking skills, puzzle and strategy games can be helpful.

### Aware of the risks

Although video games can be helpful in many ways, their risks should be recognized and controlled. Importantly, attention should be given to two aspects: content selection and disability population. As Glaubke and colleagues (34) illustrated, 90% of commercial video games contain some level of violent content. Combined with the finding reported by Excelmans et al. (36) that violent video games may lead to delinquent behavior, victimization, and alienation, gamers should be selective of the appropriate content. In addition, certain disability populations (e.g., ASD, ADHD, OCD) are more prone to overuse of video game, IGD, inattention, and aggressive behavior (103). It should be noted that we are not arguing that video games should be avoided completely for these populations. Instead, close monitoring and supervision are needed.

### Monitoring and supervising

As with many things, good things can quickly turn bad when used excessively without close monitoring and supervision. To maintain healthy video game-playing experiences, two aspects should be monitored: First, whether the purpose of playing video game is achieving the intended purpose, and second, whether the time spent playing video games is interfering with other life activities. It is important to design and implement the associated measurements when formulating the purpose for playing video games. In the context of educational video games, Padilla-Zea et al. (104) stressed the importance of continuous measurements to make sure the targeted outcomes are not derailed. Similar suggestions were made by Thompson (105) when using video games to improve the health behaviors of patients with Type II diabetes. Thompson emphasized that it is ideal for embedding the evaluation in the game if possible.

Video games can become problematic if the individual has difficulties managing other life activites. Literature has continuously emphasized the importance of close supervision to ensure the playing experience is healthy and productive (106, 107). In addition, the focus of supervision should extend beyond just playing time, frequency, and the content of video games. It is also equally important to monitor with whom the individual plays games. The effect of the company is bi-directional. For example, Osmanovic and Pecchioni (108) illustrated that video games can improve family relationships and connections when played with family members. On the other hand, when playing online video games with strangers, cyberbullying and cyber-victimization could happen (109, 110).

### Resources

In this last section, we share useful resources (see Table 2) for those interested in leveraging video games in their work with people with disabilities. Specifically, these websites and information provide detailed ideas of encouraging healthy video game habits and showcase real-life examples of how accommodations can be implemented in video games. We hope it will serve as a start and spark more discussions on this important but overlooked topic.

**Table 2:** Video game resource.

Resource	Website	Description
Ablegamers charity	https://ablegamers.org/	A community that provides resources for individuals with disabilities to engage in video games. The focus is on combating social isolation by playing games. A wealth of information about gaming specific assistive technology is included on the website. In addition, the website also provides suggestions for game developers to be more inclusive when designing the game.
Ability power	http://www.abilitypowered.com/	Created by a person with a disability who found joy in playing video games. The website provides game accessibility review. The support group format of the website offers discussion on the Discord and Steam. Ability Power also has a YouTube channel that streams their video game play.
Blind gamers	https://blindgamers.com/	A self-help website specifically created for players with visual impairments. The website has a clean look and is screen reader friendly. The website provides a platform for players to rate how accessible and enjoyable the games are.
Dager system gaming enabled	https://dagersystem.com/	Founded in 2012 by a group of video game players with disabilities. The website provides reviews of game accessibility using a four-point rating system on aspects including visual, audio and fine-motor control. A database for gaming accessibility in on the way and will be available soon.
The controller project	https://thecontrollerproject.com/	The website focuses on developing accessible controller for playing video games. Besides a library showing how the controllers can be modified to be more accessible, they also accept requests from players who are interested in customizing their controllers. A YouTube channel is available to show how the controller can be modified.
International game developers association: game accessibility special interest group	https://igda-gasig.org/	A special interest group for video game accessibility under International Game Developers Association. The group provides a channel for communication between the game developers and players with disabilities. In addition, it also provides suggestions for researchers who are interested in this topic.

## Conclusion

Video games stand to offer much to the field of rehabilitation. There are numerous benefits that educators, practitioners, and researchers can take advantage of to help those with disabilities. With educational purposes in mind, educators can leverage serious gaming design to teach students mathematic and spatial learning; with the potential to improve emotional regulation, practitioners can rely on video games to better serve their clients with psychological needs; with the cacpacity to promote self-determination and motivation, researchers can design more game-based intervention and gamify the intervention process. However, there are also a number of risks that should be accounted for when designing or implementing interventions that use video games. Certain disability populations may be more susceptible to the negative effects of video games. Due to the concerns, such as eccessive use of video games, some decide to completely avoid it. While we understand these concerns, we argue that with careful and stragtegic planning, video games can be a useful tool. With this in mind, users should strive to have a well-defined purpose, be aware of the risks, and note the importance of monitoring and supervision when utilizing video games with vulnerable populations.
